# Jarvis on Insanity

**Published:** 1844-02

**Authors:** 


// THE WESTERN JOURNAL.

New Series.—Vol. I.—No. II.

LOUISVILLE, FEBRUARY 1, 1844.

### JARVIS ON INSANITY.

"Insanity among the colored population of the Free States," is the title of an article in the January number of the American Journal of the Medical Sciences, which has been published in a separate form, and of which we are indebted for a copy to the politeness of the author. Dr. Jarvis is known to the readers of the Western Journal as the author of some valuable papers on the Lunatic Asylums of the United States. He has been industrious in collecting the statistics of insanity in this country, and the pamphlet before us brings to light some curious facts, not, indeed, in relation to insanity, but touching the character of the sixth census as it respects the prevalence of mental disorder among the different classes of our population.

The impression made by the details of the sixth census concerning the existence of insanity in this country, and especially among the two races, will be remembered. The statement, that slavery was proved by the experience of our country to be tenfold more favorable to mental health than freedom, was not likely to escape the severest examination. Dr. Jarvis has sifted the evidence on which it rests, with his characteristic patience, and the result is given in the paper before us. He finds the sixth census to abound in errors—shows that colored lunatics are set down for places where not a single individual of the African race is to be found—in a word, that the document, so far as the statistics of insanity are concerned, is "a bearer of falsehood to confuse and mislead." "In many towns *all* the colored population are stated to be insane, in very many others, two-thirds; one-half, one-fourth, or one-tenth of this ill-stared race are reported to be thus afflicted, and as if the document delighted to revel in error, every proportion of the negro population from seven-fold its whole number to less than a two-thousandth, is declared to be lunatic." Dr. Jarvis shows all this in tables which he has made out from the census.

Thus, in Shelby, New York, the total colored inhabitants is stated to be 1—the colored insane 7. The negroes in Worcester, Massachusetts, are 151 in number, 36 of whom are under 10 years of age; yet the census makes out 133 colored insane in that town. In Athens, Vermont, the black population is 2, and both are reported insane. Freetown, Leominster, Wilmington, Sterling, and Danvers, in Massachusetts, are reported by the census as destitute of a black population, but yet this queer document contrives to get two colored insane into each of these places. Plympton, in the same commonwealth, has two free blacks, according to the cansus, and twice the number of colored lunatics. Waterford in Connecticut, is still more unfortunate—her black population, amounting to 2, furnishes 7 lunatics! Providence, N. Y., has 3 colored inhabitants, of whom two are insane, and two both *deaf and dumb;* and Dryden, in the same State, reported to have no colored inhabitants, is set down as containing two colored insane, and also "two blind negroes!" But the most astonishing prevalence of insanity among the poor blacks, is reported at Washington, in Pickaway county, Ohio, where the colored inhabitants are set down in the census at 0, and the colored insane at 7!

Well may Dr. Jarvis say, in view of these errors, "we are disappointed, we are mortified." He commends the matter to the attention of Congress. He suggests that the original papers respecting the sixth census be revised, by which all the errors made in copying may be corrected. Y.

